# Risk Assessment of Triflumezopyrim and Imidacloprid in Rice through an Evaluation of Residual Data

**DOI:** 10.3390/molecules27175685

**Published:** 2022-09-03

**Authors:** Yue Zhang, Meiran Wang, Thiphavanh Silipunyo, Haizhu Huang, Qingchun Yin, Bingjun Han, Mingyue Wang

**Affiliations:** 1 Analysis and Test Center, Chinese Academy of Tropical Agricultural Sciences, Key Laboratory of Tropical Fruits and Vegetables Quality and Safety for State Market Regulation, Key Laboratory of Quality and Safety Control for Subtropical Fruit and Vegetable, Ministry of Agriculture and Rural Affairs, Hainan Provincial Key Laboratory of Quality and Safety for Tropical Fruits and Vegetables, Haikou 571101, China; 2 Plant Protection Center, Department of Agriculture, Ministry of Agriculture and Forestry, Vientiane P.O. Box 811, Laos; 3 Hainan Institute for Food Control, Haikou 570311, China

**Keywords:** triflumezopyrim, imidacloprid, risk assessment, rice

## Abstract

Triflumezopyrim, a novel mesoionic insecticide used to control planthoppers, is a potential substitute for imidacloprid. In this study, triflumezopyrim and imidacloprid residues in rice were determined using a quick, easy, cheap, effective, rugged, and safe procedure combined with ultra-high-performance liquid chromatography–tandem mass spectrometry. The limit of quantification of both triflumezopyrim and imidacloprid was 0.01 mg kg^−1^, and the average recovery values were 94–104% and 91–106%, with relative standard deviations (RSDs) of 1.1–1.4% and 2.1–3.4% (n = 5), respectively. The consumer protection level was assessed by calculating the theoretical maximum daily intake using the reported maximum residue limits of triflumezopyrim and imidacloprid. The established method was successfully applied to 200 commercial rice samples collected from four provinces in China, and their potential public health risks were assessed using triflumezopyrim and imidacloprid residues. The risk associated with triflumezopyrim and imidacloprid dietary intake was assessed by calculating the national estimated short-term intake and the acute reference dose percentage (%ARfD). The results show that the theoretical maximum daily intake (NEDI) values of triflumezopyrim and imidacloprid in different age and gender groups were 0.219–0.543 and 0.377–0.935 μg kg^−1^ d^−1^ bw, and the risk quotient (RQ) values were 0.188–0.467% and 0.365–0.906%, respectively. The acute reference dose (%ARfD) of triflumezopyrim and imidaclopridin ranged from 0.615 to 0.998% and from 0.481 to 0.780%, respectively.

## 1. Introduction

Rice is one of the world’s three major grains and serves as a staple food for almost half of the global population [1]. Rice planthoppers are the most economically important pests infesting rice in Asia [2,3] and have gained increasing public attention. Imidacloprid, a neonicotinoid insecticide, is widely used to control rice planthopper populations. However, with the increasing application of imidacloprid, pests have developed resistance. Moreover, the negative impact of this compound on pollinators, such as bees, further affects the ecological balance. Imidacloprid induces widespread disruption in the behavior of within-nest worker bees, which may contribute to their impaired growth [4,5,6]. The compound has also been reported to significantly reduce growth rate and cause an 85% reduction in the production of new queens [7]. In addition, the climbing ability of the affected bees is significantly impaired, and cell dysfunction is evident [8]. Pesticide residue levels and risk monitoring are important aspects of agricultural product quality in many countries [9,10,11,12].

**Figure 1:**
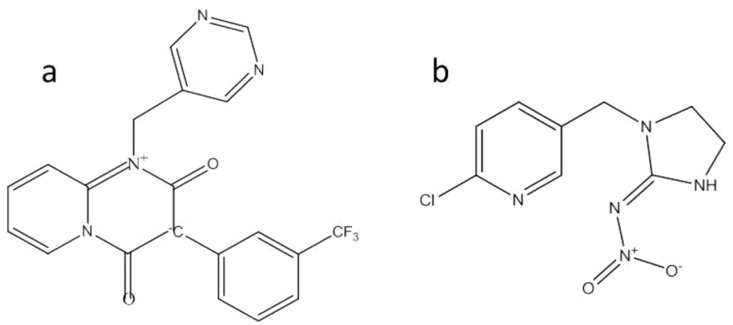
Chemical structure of triflumezopyrim (**a**) and imidacloprid (**b**).

Triflumezopyrim (chemical structure shown in Figure 1) is a novel mesoionic insecticide that possesses a pyridopyrimidinedione core that controls planthoppers without adverse effects on pollinators [13]. This class of mesoionic agents targets the nicotinic acetylcholine receptors by inducing a physiological action (unique among neonicotinoids) that prevents nerve conduction, causing the rice planthopper to lose consciousness as a result [14,15]. This insecticide controls susceptible and resistant hopper populations at low application rates, thereby conferring excellent protection to rice plants against a variety of planthoppers. Most importantly, it has been reported to have no adverse effects on pollinators. Therefore, triflumezopyrim is highly effective in controlling rice planthoppers and can replace new nicotinic insecticides, such as imidacloprid, which are associated with high resistance.

To determine if the exposure to or intake of a compound exceeds human health safety limits, toxicological endpoint values, such as allowable daily intake (ADI) and acute reference dose (ARfD), should be assessed. Such assessments reveal if the residue concentration is above the maximum residue limit (MRL) without representing a risk to the consumer [16,17,18]. Risk assessments are performed to evaluate the risks associated with consuming food contaminated with pesticides; thus, exposure assessment is a key step in calculating dietary intake risk and is based on the actual residue and consumption data for each country [19,20,21]. To the best of our knowledge, only a few studies to date have compared the health risks associated with triflumezopyrim and imidacloprid treatment in rice. It is necessary to establish a rapid, sensitive, efficient, and reliable pesticide residue detection method for rice and compare the health risks associated with triflumezopyrim and imidacloprid. Therefore, to potentially substitute imidacloprid with triflumezopyrim to control rice planthoppers, we aimed to establish an analytical method using ultra-high performance liquid chromatography–tandem mass spectrometry (UPLC-MS/MS) for the simultaneous determination of triflumezopyrim and imidacloprid residues in rice and to evaluate and compare the long-term and short-term risks posed by each of these pesticides. The results of our study provide basic data supporting the safety of triflumezopyrim application in the cultivation of rice.

## 2. Results and Discussions

### 2.1. Optimization of Extraction Conditions

**Figure 2:**
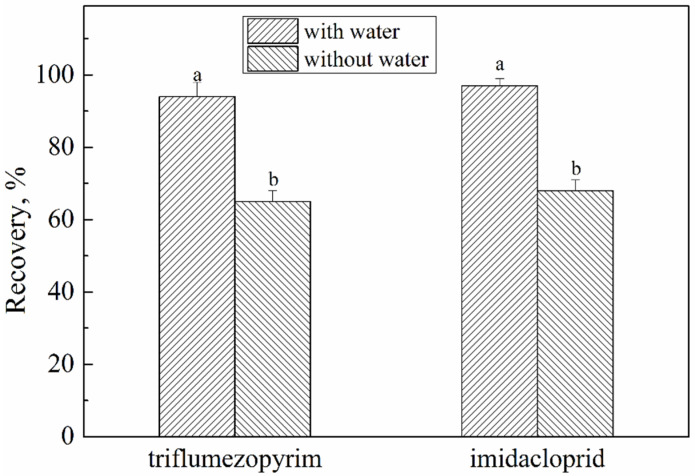
Effect of different extraction methods with and without water on recovery. The letters (a and b) indicate a significant difference at the 1% level.

In the first experiment, the extraction solvent was studied to achieve the satisfactory extraction efficiency. Considering the polarity of the target, acetonitrile was selected as the extraction solvent, which achieved a high extraction efficiency and good separation for the subsequent salting out and other purification methods. However, dry rice powder led to the poor recovery of triflumezopyrim and imidacloprid. Therefore, water was added to the rice powder samples before acetonitrile extraction. The effects of the addition of water to acetonitrile and acetonitrile on the extraction of rice samples were investigated, and the results are shown in Figure 2.

There were significant differences in the influence of the two pesticides with or without water during extraction. The extraction rate after adding water was higher than the extraction rate without adding water. When water was added to the samples, the target pesticides mixed easily with the acetonitrile, and the pesticides were extracted from the samples sufficiently. Therefore, we added water to the samples and then performed extraction with acetonitrile.

### 2.2. Optimization of Purification Conditions 

**Figure 3:**
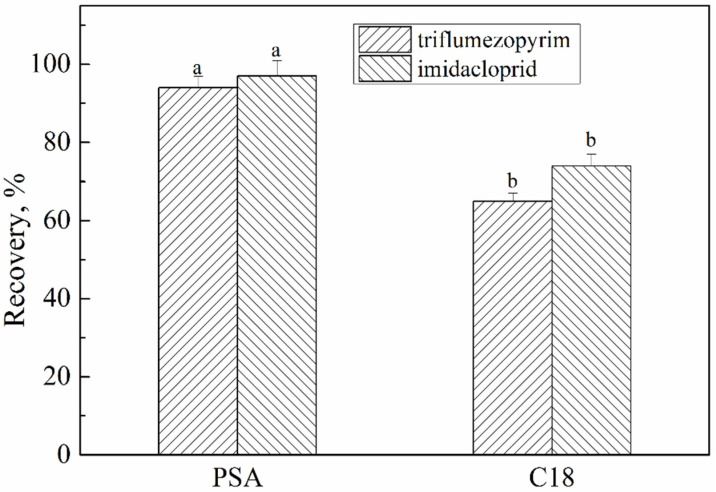
Effect of different purification sorbents on recovery. The letters (a and b) indicate a significant difference at the 1% level.

After the extraction of triflumezopyrim and imidacloprid from the rice samples, 3 mL of the acetonitrile supernatant was transferred to a mixture of different purified sorbents. Primary secondary amine (PSA) and C_18_ were selected to test the purification effects at a spiked level of 0.2 mg kg^−1^. The results are summarized in Figure 3. The recovery rate of C_18_ was approximately 70%, while the recovery rate of PSA was between 94 and 97%. PSA provided polar adsorption and weak anion exchange, which removed acid-interfering substances and polar pigments, such as fatty acids, phenols, and carbohydrates. C_18_ is often used to remove non-polar substances, such as lipids, and has an effective clean-up effect on animal-derived agricultural products with a high-fat content [22,23]. Therefore, PSA was used for further purification.

### 2.3. Method Validation

**Figure 4:**
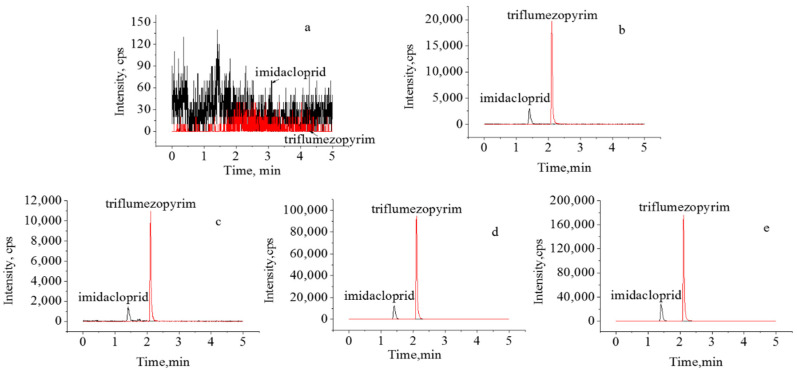
Typical LC-MS/MS chromatograms of triflumezopyrim and imidacloprid. (**a**) blank sample, (**b**) standard, (**c**) recovery at 0.01 mg/kg, (**d**) recovery at 0.1 mg/kg, (**e**) recovery at 0.2 mg/kg.

To determine the recovery and precision of the analytical method, different concentrations of mixed standard solutions were added to the blank sample matrix for all spiking experiments and matrix effect studies (shown in Table 1, Table 2 and Figure 4). The results showed that the proposed method had good linearity (concentration range: 0.005, 0.01, 0.02, 0.05, and 0.1 μg/mL), trueness, precision (repeatability and intermediate precision), and the limit of quantification (LOQ). Significant signal enhancement was observed for triflumezopyrim, whereas the signal was reduced for imidacloprid, indicating a matrix-weakening effect. Therefore, to ensure the accuracy of quantitative measurements, a matrix-matching standard solution correction method was used to compensate for the matrix effect (ME).

The accuracy was determined by the addition of known amounts of standards to the rice samples, and was calculated based on the difference in the total concentrations between the spiked and original samples. The trueness of triflumezopyrim ranged from 94 to 104% and that of imidacloprid ranged from 91 to 106% in all experiments (Table 3). Therefore, the accuracy of the proposed method was satisfactory. The method was precise, with the relative standard deviation (RSD) varying from 1.1 to 1.4% for triflumezopyrim and from 2.1 to 3.4% for imidacloprid. In routine analysis, the acceptable range of precision was less than 20%. These results demonstrate that the method developed in this study provides satisfactory reproducibility and good analytical performance.

### 2.4. Pesticide Residues in Rice Samples

**Figure 5:**
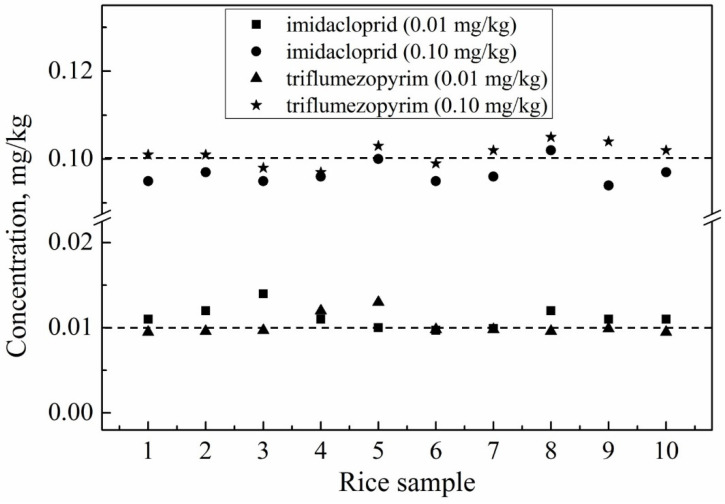
Residues of triflumezopyrim and imidacloprid in positive rice samples.

The validated method was used to analyze the two target pesticide residue levels in 200 rice samples. The results showed that there were no residues in the 200 tested samples (data not shown). Samples of triflumezopyrim and imidacloprid were then spiked at a concentration of 0.01 and 0.10 mg/kg into rice, which were regarded as the positive samples for the real sample test. The analytical results are presented in Figure 5. Considering the maximum risk to human health, the supervised trial median residue (STMR) and the maximum residue level (HR) values of triflumezopyrim and imidacloprid were collected from the report of the Joint FAO/WHO Meeting on Pesticide Residues (JMPR). As shown in Table 3, the STMRs of triflumezopyrim and imidacloprid at 0.05 and 0.086 mg/kg, respectively, were used for the long-term intake assessment, and the HRs of triflumezopyrim and imidacloprid at 0.05 and 0.16 mg/kg, respectively, were used for the short-term intake assessment.

### 2.5. Risk Assessment

#### 2.5.1. Long-Term Intake Assessment

**Figure 6:**
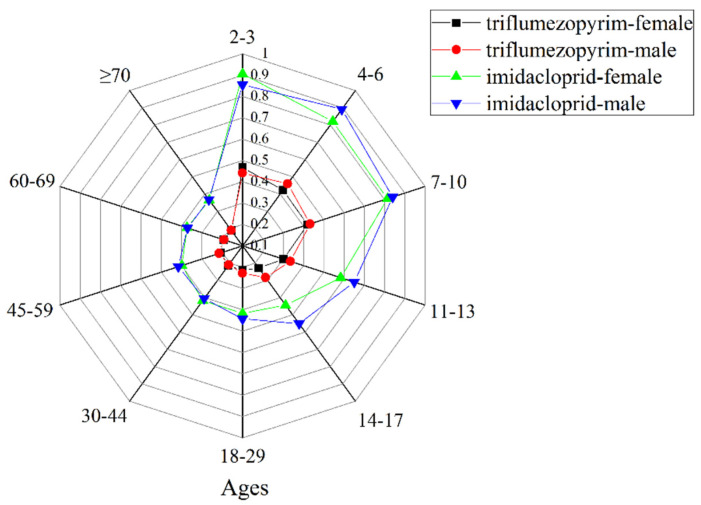
Risk quotient (RQ) of triflumezopyrim and imidacloprid in rice for the different ages and sexes.

We collected the acceptable daily intake (ADI), acute reference dose (ARfD), STMR, and HR values of triflumezopyrim [24] and imidacloprid [25] from the JMPR report, which are shown in Table 3. These data were used for the comparison of risk assessments of the two target pesticides. The risk assessment results of chronic dietary exposure to the two pesticides were collected at the first stage of our comparison, and the results are shown in Table 4 and Figure 6. The theoretical maximum daily intake (NEDI) values of triflumezopyrim and imidacloprid were 0.219–0.543 and 0.377–0.935 μg kg^−1^ d^−1^ bw, respectively, and their risk quotients (RQs) were 0.188–0.467% and 0.365–0.906%, respectively. The RQ values of the two pesticides were less than 100%, indicating that risk is acceptable with a high protection level and implying that the pesticide will not constitute a health threat in the long term. The RQ of triflumezopyrim was slightly lower than that of imidacloprid. Triflumezopyrim provides a high level of protection to consumers in terms of the risk associated with chronic ingestion. Table 4 shows that the RQ of males was lower than that of females. Meanwhile, with an increase in age, RQ decreased, indicating that the risk decreased.

#### 2.5.2. Short-Term Intake Assessment 

**Figure 7:**
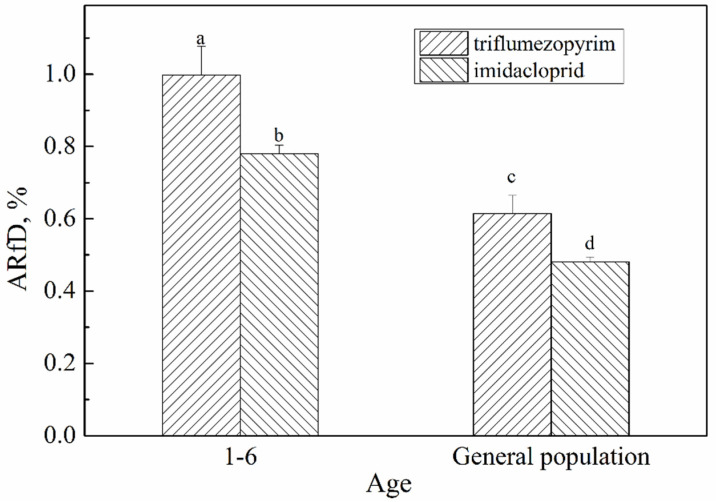
%ARfD of triflumezopyrim and imidacloprid in rice for different ages. The letters (a, b, c and d) indicate a significant difference at the 1% level.

We used the rice consumption data for the Chinese population, which are divided into data related to the general population and data of children 1–6 years old, as recommended by the GEMs/Food project to calculate short-term dietary risk assessment. The results are presented in Table 5 and Figure 7. The %ARfD of triflumezopyrim and imidacloprid ranged from 0.615 to 0.998% and from 0.481 to 0.780%, respectively. These %ARfD values were much lower than 100%, indicating that the risk of acute dietary intake was acceptable. However, the %ARfD for children aged 1–6 was higher than that of the general population, indicating that the risk is higher in children than in adults.

## 3. Materials and Methods

### 3.1. Materials and Reagents

A high-purity (99.4%) pesticide standard of triflumezopyrim was obtained from DuPont Crop Protection (Wilmington, DE, USA), and a high-purity standard of imidacloprid (1000 μg/mL) was purchased from the Environmental Quality Supervision and Testing Center, Ministry of Agriculture and Rural Affairs (Tianjin, China). HPLC-grade acetonitrile and methanol were obtained from Thermo Fisher Inc. (Waltham, MA, USA), and HPLC-grade ammonium acetate was obtained from Sigma-Aldrich (St. Louis, MO, USA). PSA and C_18_ were purchased from Agela Technologies (Tianjin, China). Syringe filters (nylon, 0.22 μm) were purchased from ANPEL Laboratory Technologies Inc. (Shanghai, China). A stock solution of triflumezopyrim was prepared at 1000 μg/mL in methanol and stored at −20 °C until analysis.

### 3.2. Sample Acquisition 

The 200 rice samples used in this study were procured from different regions in China. These were purchased from wholesale markets and supermarkets in Zhejiang, Shandong, Beijing, and Hainan Provinces. The sampling locations of all the rice were representative, and included the southern and northern planting bases. After sampling, the samples were ground into powder, placed in sealed pockets, and stored in a refrigerator at −20 °C prior to analysis.

### 3.3. Sample Preparation 

Pretreatment was conducted using a quick, easy, cheap, effective, rugged, and safe method [26] with minor modifications. Rice powder samples (5 g) were placed in 50 mL centrifuge tubes. To these, 5 mL of water and 10 mL of acetonitrile were added to extract the target compounds. Subsequently, the solution was homogenized for 2 min, after which 5 g of sodium chloride was added. The mixture was vortexed at 3000 rpm for 1 min, followed by centrifugation at 8000 rpm for 5 min. The supernatant (5 mL) was transferred to a 15 mL centrifuge tube containing 150 mg PSA and 900 mg magnesium sulfate. The mixture was then vortexed and centrifuged at 8000 rpm for 5 min. The supernatant was filtered through a 0.22 μm sieve and collected for further testing.

### 3.4. UPLC-MS/MS Analysis 

Chromatographic analysis was performed using a Waters Acuity UPLC and triple quadrupole mass spectrometer (AB SCIEX, Framingham, MA, USA) equipped with an electrospray ionization source in the positive ionization mode. Chromatographic separation was achieved using a C_18_ column (100 mm × 2.1 mm dimensions, 1.7 µm particle size) with a binary mobile phase comprising solvent A (0.1% formic acid) and B (100% methanol) at 35 °C. The mobile phase gradient was set as follows: 0 to 0.1 min, 90% A; 0.1 to 1.0 min, 90 to 5% A; 1.0 to 3.0 min, 5% A; 3.0 to 3.1 min, 5 to 90% A; and 3.1 to 5.0 min, 90% A. The flow rate was 0.25 mL/min, and the injection volume was 5 μL. The multiple reaction-monitoring parameters are listed in Table 6.

### 3.5. Method Validation

The analytical method was validated using a conventional approach, including the determination of selectivity, linearity, recovery, limit of detection (LOD), LOQ, ME, and repeatability. The parameters of linearity, including the slope, intercept, and determination coefficient, were calculated based on a five-point calibration curve. An isotopically labeled standard was used to eliminate ME as reported previously [27]. Such compounds can help in the correction of signal deviation because they have the same chemical properties and retention times as non-labeled compounds. However, isotopically labeled internal standards were not available for all the analytes. Therefore, we used a matrix-matching standard for quantitative analysis.

Calibration curves were obtained by plotting the peak area (y) of the analyte versus its concentration (x) in the matrix-matched standard solution (matrix-matched calibration). A standard solution was used throughout the analysis to check for possible signal fluctuations.

ME is a major concern in food analysis, and most sample matrices are assessed by comparing the peak area ratios of matrix-matched calibration curves with that of solvent-based calibration curves [26,28]. The ME was calculated using Equation (1) with the peak area of the standard in the solvent (*A_solvent_*) and the peak area of the standard in the matrix (*B_matrix_*) [29,30,31,32].

(1) $$\;ME=\frac{B_{matrix}-A_{solvent}}{A_{solvent}}\times 100\%$$

where *B_matrix_* is the average area of the analyte curve, spiked in the extracted matrix after the extraction procedure, and *A_solvent_* is the average area for the same concentration of analyte in the neat solution. Therefore, a negative result indicates a suppressed signal, and a positive result indicates an enhanced analyte signal.

The LOD was three times the signal/noise ratio [33]. The LOQ was set at the lowest validated level with acceptable trueness (70–120%) and precision (relative standard deviation (RSD) < 20%).

The accuracy of the measurement method was confirmed by determining trueness and precision using a routine recovery assay at three levels of fortification (0.01, 0.10, and 0.20 mg/kg) and replicated five times. The precision of the method was determined by assessing its repeatability and reproducibility and was expressed using RSD% [34,35]. The target compounds were extracted and purified using the aforementioned sample extraction procedures.

### 3.6. Statistical Calculations 

Dietary exposure and risk entropy values are primarily used to assess exposure pathways and calculate possible dose exposure levels to determine actual and expected exposure levels among potentially sensitive populations. Based on the dietary structure of different populations in China and pesticide residue data from this study, we conducted a risk assessment of chronic and acute dietary exposure to pesticides used for rice.

### 3.7. Long-Term Intake Assessment 

The dietary structure and food consumption of the population were determined, and the maximum daily intake of certain pesticides was calculated according to the *STMR*. Dietary exposure and risk were calculated as follows:

(2) $$NEDI=\sum^{\;}\left(F_{i}\times STMR_{i}\right)/bw$$

(3) $$\mathrm{RQ}=\frac{NEDI}{ADI}\times 100\%$$

where *NEDI* is the theoretical maximum daily intake (μg kg^−1^ d^−1^ bw), *F_i_* is the food intake (g·d^−1^), *STMR_i_* is the supervised trial median residue (mg kg^−1^), *bw* is the body weight of different age groups (kg) (Table 4), RQ is the risk quotient (%), and *ADI* is the acceptable daily intake (mg kg^−1^ bw).

When the RQ is less than 100, the risk is acceptable with a high protection level, meaning that the pesticide will not constitute a health threat in the long term. When the RQ is higher than 100, indicating a low level of protection, consumer health is at an unacceptable risk. The higher the RQ value, the greater the chronic exposure risk [36,37,38].

### 3.8. Short-Term Intake Assessment 

The concentration of residue in a composite sample (raw or processed) reflects a large portion of the commodity. Single-day consumption data for high-end (97.5th percentile) consumers were required to calculate the national estimated short-term intake (*NESTI*). Considering the variability from unit to unit in the composite sample, the *NESTI* assumed by the model according to the unit weight (URAC) was <25 g.

(4) $$NESTI=\frac{LP\times HR}{bw}$$

For rice, the available composite residue data reflect the residue levels in a meal-sized portion of the product (commodity unit weight, <25 g) in cases where the commodity is well-mixed during processing [19]. In this study, Ue < 25 g; therefore, *NESTI* was calculated using Equation (4).

(5) $$\%ARfD=\frac{NESTI}{ARfD}\times 100\;$$

where *LP* is the weight of a large serving of food (kg), *HR* is the maximum residue level (mg kg^−1^), and *ARfD* is the acute reference dose (mg kg^−1^·bw). When the %*ARfD* ≤ 100%, the risk of acute dietary intake is considered acceptable. The smaller the *ARfD* value, the lower the risk [39]. In contrast, when %*ARfD* > 100%, the risk is considered unacceptable.

## 4. Conclusions

We developed and validated a rapid, sensitive, and selective method for the detection of triflumezopyrim and imidacloprid residues in rice and analyzed 200 rice samples from different markets.

In both long- and short-term risk assessments, the risk values were much lower than 100%, indicating that the risk was acceptable. With an increase in age, the risk values decreased. The RQ of triflumezopyrim was slightly lower than that of imidacloprid in the long-term intake assessment; therefore, triflumezopyrim is a potential substitute for imidacloprid to control planthoppers in rice.

## Figures and Tables

**Table 1:** Average recoveries and RSDs at three spiked levels.

Compounds	Spiking Levels/(mg/kg)	Average Recovery/%	RSD/%	LOQ/(mg/kg)
imidacloprid	0.01	106	2.1	0.01
	0.10	91	2.6	
	0.20	97	3.4	
triflumezopyrim	0.01	104	1.1	0.01
	0.10	101	1.4	
	0.20	94	1.4	

**Table 2:** Method validation parameters.

Compounds	Matrix	Regression Equation	Correlation Coefficients	ME/%
imidacloprid	solvent	y = 1,963,648x − 3902	0.9999	-
	rice	y = 1,176,517x − 449	0.9980	−37.3
triflumezopyrim	solvent	y = 1,617,540x + 211	0.9998	-
	rice	y = 5,291,693x − 1059	0.9992	224.2

**Table 3:** Risk assessment data according to the JMPR report.

Compounds	ADI, mg/kg bw	ARfD, mg/kg bw	STMR, mg/kg	HR, mg/kg
imidacloprid	0.06	0.4	0.05	0.05
triflumezopyrim	0.2	1	0.086	0.16

**Table 4:** Long-term intake assessment at the different age groups.

Age	Gender	bw/(kg)	F_i_/(g d^−1^)	NEDI/(μg kg^−1^ d^−1^ bw)		RQ/(%)	
				Imidacloprid	Triflumezopyrim	Imidacloprid	Triflumezopyrim
2–3	Male	13.2	135.5	0.883	0.513	0.855	0.441
	Female	12.3	133.7	0.935	0.543	0.906	0.467
4–6	Male	16.8	179.7	0.920	0.535	0.891	0.460
	Female	16.2	159.5	0.847	0.492	0.820	0.423
7–10	Male	22.9	230.8	0.867	0.504	0.840	0.433
	Female	21.7	212.0	0.840	0.488	0.814	0.420
11–13	Male	34.1	266.2	0.671	0.390	0.651	0.336
	Female	34.0	238.4	0.603	0.351	0.584	0.302
14–17	Male	46.7	308.7	0.568	0.331	0.551	0.284
	Female	45.2	240.7	0.458	0.266	0.444	0.229
18–29	Male	58.4	309.6	0.456	0.265	0.442	0.228
	Female	52.1	260.9	0.431	0.250	0.417	0.215
30–44	Male	64.9	316.2	0.419	0.244	0.406	0.210
	Female	55.7	278.6	0.430	0.250	0.417	0.215
45–59	Male	63.1	314.9	0.429	0.250	0.416	0.215
	Female	57.0	272.8	0.412	0.239	0.399	0.206
60–69	Male	61.5	274.0	0.383	0.223	0.371	0.192
	Female	54.3	242.9	0.385	0.224	0.373	0.192
≥70	Male	58.5	258.3	0.380	0.221	0.368	0.190
	Female	51.0	223.5	0.377	0.219	0.365	0.188

**Table 5:** Short-term intake assessment of triflumezopyrim and imidacloprid.

Age	bw/(kg)	LP/(g d^−1^)	Ue/(g)	NESTI/(μg kg^−1^ d^−1^, bw)		%ARfD/(%)	
				Imidacloprid	Triflumezopyrim	Imidacloprid	Triflumezopyrim
1–6	16.1	1004.28	<25	3.119	9.980	0.780	0.998
General population	53.2	2046.23	<25	1.923	6.154	0.481	0.615

**Table 6:** MS/MS parameters for the analysis.

Compounds	Ions	Declustering Potential/V	Collision Energy/V
imidacloprid	256.2/175 * 256.2/209	69	25.2 18.4
triflumezopyrim	399.1/278.1 * 399.1/121	120	40 50

* Quantitative ion.

## Data Availability

All data presented in the present research are available on request from the corresponding author.
